# Unsupervised Identification of Driving Styles from Naturalistic Driving Data Through a Context-Normalized Framework

**DOI:** 10.3390/s26134309

**Published:** 2026-07-07

**Authors:** Cunzhi Xu, Reuben S.K. Agbozo, Liang Huang, Zheng Zhang, Tao Peng, Renzhong Tang

**Affiliations:** 1 State Key Laboratory of Fluid Power Components and Mechatronic Systems, School of Mechanical Engineering, Zhejiang University, Hangzhou 310058, China; 11825021@zju.edu.cn (C.X.); 11925098@zju.edu.cn (Z.Z.); tangrz@zju.edu.cn (R.T.); 2 Institute of Manufacturing Technology and Systems Engineering, School of Mechanical Engineering, Zhejiang University, Hangzhou 310058, China; reuben@zju.edu.cn (R.S.K.A.); 11925108@zju.edu.cn (L.H.)

**Keywords:** driving style, unsupervised deep learning, constrained autoencoder, self-attention mechanism, driving behavior, context normalization

## Abstract

**Highlights:**

**What are the main findings?**

- An unsupervised context-normalization framework was developed to identify driving styles from unlabeled naturalistic CAN-bus data.
- WLTC-anchored DACs enabled comparable style characterization, and the identified styles showed distinct operating characteristics and historical accident probabilities.

**What are the implications of the main findings?**

- The framework provides an interpretable basis for analyzing large-scale unannotated driving datasets.
- The results support driver behavior analysis and personalized ADAS applications.

**Abstract:**

Identifying driving styles is essential for personalizing driving assistance systems and enhancing intelligent transportation services. However, existing approaches predominantly rely on experience-driven feature engineering and annotated data, limiting objectivity and hindering the exploitation of unlabeled naturalistic driving datasets. To address these limitations, this paper proposes an unsupervised framework for driving style identification from naturalistic driving data through context normalization. A Constrained Convolutional Autoencoder (CCAE) integrated with a global self-attention mechanism is developed to map unlabeled driving sequences onto a standardized dynamic reference defined by the Worldwide Harmonized Light Vehicles Test Cycle (WLTC). This process extracts Driving Adaptability Characteristics (DACs) as WLTC-anchored latent representations that characterize normalized driver-specific response patterns across heterogeneous naturalistic contexts. To ensure feature robustness, frequency-domain refinement is applied to eliminate high-frequency noise. The extracted DAC sequences are subsequently partitioned into distinct driving styles using a kernel-mapped clustering algorithm. To evaluate the external relevance and physical interpretability of the identified styles, actual vehicle accident records and raw CAN-bus feature backtracking are introduced as validation evidence. The results show that the identified driving styles exhibit different historical accident probabilities. The proposed CCAE model achieves clearer cluster-level differentiation than traditional feature engineering and unconstrained deep learning models, and the ablation analysis confirms the contribution of the WLTC-based constraint. These findings indicate that the context-normalization framework can extract interpretable and externally relevant driving style representations from unlabeled naturalistic data.

## 1. Introduction

Understanding and analyzing driving styles is essential for advancing road safety, improving driver behavior, and enhancing intelligent transportation system (ITS) services. Driving style generally refers to the habitual and characteristic manner in which a driver controls a vehicle, involving the manipulation of the accelerator, brake, gear shift, and steering wheel to align the vehicle’s performance with individual driving intentions and expectations [1]. This personalized mode of driving is shaped by distinct tendencies, preferences and decision-making propensities [2,3]. Different driving styles significantly impact fuel economy, vehicle maintenance, and, most importantly, transportation safety. According to a World Health Organization (WHO) global status report, over 1.2 million people are killed annually on the roads, with driver behavior as one of the key contributing factors [4]. Additionally, official reports show that driver-related factors play a dominant role in traffic accidents, accounting for approximately 95% of reported cases [5]. These findings highlight the critical role of driving style in road safety outcomes. Therefore, reliable methods for identifying driving styles are urgently needed to support proactive safety interventions and improve overall transportation safety [6,7,8].

To meet this need, various data sources have been explored to analyze driver behavior, including questionnaire survey, psychological and physiological signals, video monitoring, and on-board sensors. Questionnaire-based surveys and psychometric inventories have been used to characterize habitual driving styles, perceived safety attitudes, and driving aggressiveness, but they mainly rely on self-reported information and are less suitable for continuous objective monitoring in naturalistic driving studies [1]. Physiological and psychological signals, such as heart rate, eye movement, or brain activity, have been used to infer driver behavior but often require intrusive equipment, making them impractical for naturalistic driving studies [9,10,11,12]. Similarly, camera-based approaches are developed to monitor driving behaviors, such as drowsy driving [13], lane change [14,15], and car following [16], which can be susceptible to external factors such as lighting and visibility. Alternatively, data collected through the Vehicle Controller Area Network (CAN) bus provides a non-intrusive, practical, and effective means for real-time monitoring of driving behavior, i.e., lane departures [17], distraction [18], car following [19], integrated driving behavior identification [20], making it a valuable source for driving style analysis in real-world settings.

The advancement of various feature extraction techniques has significantly enhanced the understanding and analysis of driving behavior characteristics. These techniques include the following: (1) simulation and mathematical modeling [1,21]; (2) traditional machine learning methods [22,23,24]; (3) supervised deep learning methods [13,25,26,27,28]; (4) unsupervised deep learning methods [29,30,31,32,33,34]. While these methods differ in model structure and application scope, many traditional machine learning and supervised deep learning approaches rely heavily on expert-designed features or labeled data, which limits their scalability for large-scale unlabeled CAN-bus datasets collected in real-world driving environments. In contrast, unsupervised methods, such as autoencoders, offer the potential for feature extraction without the need for labelled data, making them particularly promising for analyzing driving behavior in diverse real-world conditions. However, naturalistic driving data are strongly affected by heterogeneous traffic, road, and operational contexts, and the extracted features may therefore contain both driver-specific behavioral traits and scenario-induced variations. Consequently, robust feature extraction methods are needed not only to reduce label dependence, but also to obtain comparable driving style representations by mitigating context-driven interference.

Effective classification and identification of driving styles play a key role in revealing the practical applications of driving behavior analysis, such as risk-related evaluation and personalized driver assistance. Existing approaches often rely on predefined categories, such as aggressive or non-aggressive [35,36], with validation based on expert-derived safety parameters like acceleration or braking intensity. While supervised methods depend on labelled data from domain experts, unsupervised approaches aim to discover patterns directly from data but still require expert input for further analysis [37,38]. This reliance on expert knowledge introduces potential biases and limits scalability. Therefore, a robust and scalable framework for identifying driving styles from unlabeled naturalistic data is needed, especially one that can derive comparable representations under heterogeneous driving contexts and evaluate the identified styles using independent external evidence.

To address these limitations, this study proposes an unsupervised context-normalization framework for identifying driving styles from multidimensional CAN-bus data. By integrating deep learning techniques with a standardized dynamic reference, this framework is designed to reduce context-driven interference and extract comparable driving style representations across heterogeneous naturalistic conditions. Specifically, the proposed method extracts drivers’ Driving Adaptability Characteristics (DACs) under a normalized reference context, providing a data-driven and interpretable basis for personalized ITS applications and usage-based insurance. Actual vehicle accident records are further introduced as an external benchmark for evaluating the association between the identified styles and historical accident probabilities. The main contributions of this paper can be summarized as follows:

- Development of a context-normalization architecture: We propose a novel Constrained Convolutional Autoencoder (CCAE) that incorporates a global self-attention mechanism. By employing the WLTC standard speed profile as a standardized reference constraint, the model anchors the speed-related latent representation and extracts DACs as comparable driver-specific response features without the need for manual labels.
- Establishment of a robust feature refinement and clustering methodology: To handle the non-stationary nature of naturalistic driving data, we introduce an FFT-based low-pass filtering mechanism combined with a kernel-mapped clustering algorithm. This approach preserves the low-frequency structure of DAC representations, reduces high-frequency disturbances, and accommodates temporal elasticity caused by driver reaction delays.
- External validation and physical interpretability analysis of the identified driving styles: We evaluate the identified driving styles using actual vehicle accident records and raw CAN-bus feature backtracking. The results show that the identified styles exhibit different historical accident probabilities and distinguishable vehicle-level operating characteristics, providing association-level external validation and physical interpretability evidence for the proposed framework.

The remainder of this paper is organized as follows. Section 2 summarizes the related works. Section 3 details the methodology, including the physical architecture of the CCAE, frequency-domain refinement, and the kernel-based unsupervised clustering algorithm. The experimental results, comprising model training performance, latent space topological consistency validation, and risk validation using historical accident records, are presented in Section 4. Section 5 demonstrates the superiority and robustness of the proposed framework through baseline model comparisons and a sensitivity analysis of the frequency-domain truncation ratio. Finally, Section 6 provides the conclusion.

## 2. Literature Review

This section describes the related works in three aspects, associated data in driving which reflects driving behavior features, driving behavior analysis, driving style identification.

### 2.1. Associated Data in Driving

Table 1 demonstrates the diverse data and equipment used to collect data in existing studies. In the context of driving, diverse data refers to a wide range of information collected or measured from various sources and sensors to analyze and understand driving behavior. This includes physiological signals, such as breath measurements and EEG signals, as well as data captured from onboard cameras, radar systems, and vehicle sensors connected to the Controller Area Network (CAN) bus. Thence, physiological signals have been explored as a potential method for classifying driving styles and behavior. For instance, a breath sensor was used to measure electric currents of positively or negatively charged water clusters in breath for detecting drunk and drowsy driving [9]. Also, the electroencephalography (EEG) signals were used to diagnose the stress and mental states of drivers while driving [10,11]. Kong et al. [12] discovered that different psychological characteristics of different perceived distances and expected safety distances lead to various car-following strategies. However, physiological and psychological measurements often require dedicated wearable or contact-based devices, which may cause discomfort and limit their practicality in large-scale naturalistic driving studies.

Furthermore, onboard camera and radar data has also shown promise for analyzing driving behavior. For example, Ed-Doughmi et al. [13] created experiment using a sequence of the driver’s face frames recorded by on-board camera to analyze and anticipate driver drowsiness. Griesbach et al. [14] obtained lane changes information via video recordings and used an echo state network with RNN to predict driver’s lane-changing driving behaviors. Radar data has also been utilized to identify dangerous driving behaviors. For instance, an identification model of dangerous driving behaviors was built by using vehicle-mounted radars and cameras to obtain movement state information of the vehicles around the host vehicle and lane line distance data [16]. Nevertheless, many studies have shown that the quality of data collected via on-board cameras can easily be influenced by ambient lights in practical applications. Additionally, extra cameras and radars may be a burden for regular drivers. So, while physiological, camera and radar data have pros and cons for classifying driving styles, more robust methods of collecting driving behavior data under real driving conditions are needed.

With the developments of smart sensors, there are now sensors connected in vehicle CAN-bus that can accurately collect various vehicle signals such as gear signals, steering wheel signals, pedal signals. These data can be used to analyze driving behaviors. To illustrate, McDonald et al. [17] used a random forest algorithm to analyze steering wheel angle data for detecting drowsiness-related lane departures. A random forest classifier was employed to detect driver distraction with vehicle data such as speed, acceleration, brake and other data, reached a good result [18]. Wang et al. [19] combined vehicle CAN-bus data and car-following data to study and predict a driver’s car-following behavior. CAN-bus data were proved to be able to identify different driving behaviors [20]. Compared with physiological, camera, and radar data, CAN-bus data are non-intrusive, readily available from production vehicles, and suitable for long-term naturalistic driving studies. However, CAN-bus data mainly describe vehicle motion and control states, while road type, traffic density, weather, and other external context variables are often unavailable. This limitation further motivates the need for feature extraction methods that can derive comparable driving style representations under heterogeneous naturalistic contexts.

### 2.2. Driving Behavior Analysis

Analyzing the features of driving behavior is an essential process for driving style classification. Generally, driving behavior analysis considers how drivers control the vehicle under different operating conditions, including speed choice, acceleration and braking patterns, steering behavior, risk tendency, and compliance with traffic rules. Early studies often relied on mechanism-based or questionnaire-based behavioral analysis. Allen et al. [21] developed a driver/vehicle simulation modeling framework, providing an interpretable way to describe driver–vehicle interaction mechanisms. In parallel, Yang et al. [1] investigated the relationship among driving skill, driving behavior, and driving aggressiveness through questionnaire survey, factor analysis, clustering, and ordinal regression. These studies provide useful explanations of driving behavior mechanisms or psychological traits, but they usually depend on predefined assumptions, manually designed variables, or self-reported information.

Traditional machine learning methods have also contributed to driving behavior feature analysis. For instance, Ma et al. [22] used principal component analysis (PCA) to select factors with the greatest impact on turning, acceleration and deceleration. These factors were shown to indicate the differences between people with different driving styles. Sun et al. [23] used PCA to extract 7 variables from the original variables about drunk driving and reached accuracy of 92.01% in recognizing drunk driving behaviors in later experiment. Deng et al. [24] developed an approach for predicting human driving behaviors based on hidden Markov Model (HMM), and proved its effectiveness on experiment data from real human driving behaviors. Although these methods are relatively interpretable and computationally efficient, their performance depends heavily on hand-crafted variables and low-dimensional statistical features. As naturalistic driving data become larger, more heterogeneous, and more temporally complex, such feature engineering may be insufficient to capture long-term behavioral patterns.

Over the years, deep learning has gained popularity in the study of driving behavior due to due to its effectiveness in learning driving behavior features for detection and prediction. This is evident in how Shahverdy et al. [25] used it to train a 2D Convolutional Neural Network (CNN) on images constructed from driving signals, and the model proved its effectiveness on experiment of detecting the driving behavior. Jia et al. [39] proposed IDE-Net, a deep learning framework that uses multi-task learning and spatial-temporal encoding to automatically extract interactive driving events and patterns from vehicle trajectory data. S. Chen et al. [26] developed a transfer-learning-based driving behavior recognition method, in which a CNN model trained with multi-source online car-hailing data was fine-tuned using limited natural driving samples from heavy-duty freight vehicles to recognize acceleration, deceleration, turning, lane changing, and lane keeping behaviors. Also, Wei et al. [27] proposed a hybrid neural network model based on Recurrent Neural Network (RNN) and Fully Connected (FC) Neural Network to predict lane-changing behavior, the prediction accuracy was up to 93.5%. Ed-Doughmi et al. [13] applied an RNN-based model to driver face-frame sequences for driver drowsiness analysis and anticipation. Additionally, Zou et al. [28] proposed a vehicle acceleration prediction framework that combines MHMM-based driving behavior semantic segmentation with LSTM and GRU prediction models, showing that considering driver-behavior heterogeneity can improve acceleration prediction. Jabbar et al. [40] further proposed a hybrid 1D CNN–LSTM Attention model with few-shot learning for CAN-bus-based driver identification, showing the potential of deep temporal models in recognizing individual driving patterns from limited labeled samples. Malik et al. [41] applied machine learning and deep learning techniques, including CNN, Optimized Spectral Neural Classification, and Fuzzy Logical Feature Selection, to OBD-based driving behavior classification and prediction. These supervised deep learning methods can learn richer representations than traditional feature engineering, but they generally require labeled training data, which are costly to obtain and difficult to scale for large naturalistic CAN-bus datasets.

To reduce the dependence on labeled data, unsupervised deep learning approaches have emerged as an attractive alternative, allowing for the discovery and extraction of driving behavior patterns without the need for prelabeled data. Also, Zhao et al. [29] managed to predict speed and steering angle based on Deep Belief Network (DBN). Yang et al. [30] further proposed an improved DBN model, MSR-DBN, which integrates a multi-target sigmoid regression layer with DBN to predict front-wheel angle and speed based on historical ego-vehicle states, surrounding-vehicle information, and driver operations. These studies demonstrate the applicability of DBN-based models to driving behavior prediction, but DBN structures are relatively limited in extracting complex temporal representations compared with more flexible neural representation models. Autoencoders provide another unsupervised representation learning strategy and have strong potential for nonlinear dimensionality reduction and feature extraction [42,43]. For example, Nugroho [31] used autoencoder to extract cleaner and more distinguishable features from fire and smoke figures for a fire detection system. Keser and Töreyin [32] used an autoencoder to reduce the dimensionality of feature vectors for object recognition while preserving discriminative information. Kong et al. [33] evaluated deep convolutional autoencoders as generic feature extractors for seismological waveform analysis and showed that their effectiveness depends on the similarity between the encoded features and the target task. In vehicle-related applications, Chakraborty et al. [34] proposed a structural-attention-based recurrent variational autoencoder for highway vehicle anomaly detection, showing that VAE-based sequence models can learn normal vehicle behavior patterns and identify abnormal trajectories. These studies indicate the potential of autoencoder for learning driving behavior features.

Overall, existing driving behavior analysis methods have evolved from expert-driven modeling and hand-crafted statistical features to deep representation learning. However, two challenges remain for naturalistic driving style identification: first, many methods still rely on labeled data or expert-designed features; second, driving behavior features extracted from real-world CAN-bus data are often entangled with heterogeneous traffic, road, and operational contexts. These limitations motivate the development of an unsupervised feature extraction framework that can learn comparable driving style representations under context variability. The related approaches are summarized in Table 2.

### 2.3. Driving Style Identification

After driving behavior features are extracted, drivers can be further classified into different driving styles. Existing studies have defined driving styles under different category systems and application purposes. A common strategy is to use predefined style categories. For example, Johnson and Trivedi [35] identified aggressive and non-aggressive driving styles, while Zylius [36] distinguished aggressive and safe driving styles. Shahverdy et al. [25] classified driving samples into multiple predefined categories, including normal, aggressive, distracted, drowsy, and drunk driving. These studies demonstrate that predefined style categories are useful for distinguishing typical behavioral tendencies, but their classification results usually depend on labeled samples or experimentally defined behavior categories.

Another group of studies has identified driving styles for safety-related driver assistance and vehicle-control applications. K. Yang et al. [44] classified driving styles from the perspective of real-time traffic safety by combining clustering and decision-tree methods. Guan et al. [45] classified drivers into conservative, normal, and aggressive styles and incorporated the identified styles into a personalized steering feel control strategy. Lyu et al. [15] identified aggressive, normal, and conservative driving styles and used the style information to improve lane-change prediction. Wang et al. [46] divided driving styles into cautious, relatively cautious, relatively aggressive, and aggressive types based on lane-change safety and validated the classification results using lane-change safety judgment parameters. Guo et al. [47] classified driving styles into three types and incorporated them into an adaptive optimal control strategy for plug-in hybrid electric vehicles. Lin et al. [48] used a fuzzy expert algorithm to identify driving styles for fuel-economy-oriented adaptive control. These studies show that driving style identification can support personalized assistance, warning, control, and energy-management applications, but the validation of style categories often relies on task-specific surrogate indicators such as lane-change safety, steering response, or fuel economy.

Unsupervised and hybrid methods have also been used to reduce direct dependence on manually assigned style labels. Mohammadnazar et al. [37] classified driving styles into aggressive, normal, and calm types from observed speed and longitudinal/lateral acceleration using unsupervised machine learning. Cai et al. [38] combined unsupervised clustering and subjectively voting methods to classify driving styles into three types: aggressive, normal and conservative. Guo et al. [49] classified driving styles into aggressive, stable, and conservative types by first generating pseudo-labels through multiple clustering algorithms and then applying an ensemble learning strategy. These studies enrich the category systems and modeling strategies for driving style identification, but many of them still require expert interpretation, predefined style labels, subjective questionnaires, or clustering-derived pseudo-labels.

Overall, existing driving style identification studies have provided useful category definitions and application-oriented validation strategies. Nevertheless, many methods still depend on predefined style labels, expert interpretation, or surrogate indicators such as acceleration, braking intensity, lane-change safety, and fuel economy. These validation strategies are valuable but may limit scalability and objectivity when applied to large-scale unlabeled naturalistic driving data. Moreover, relatively few studies introduce independent real-world records to examine whether the identified driving styles are externally associated with practical outcomes. These limitations motivate the development of an unsupervised framework that can identify driving styles from naturalistic CAN-bus data and evaluate the identified styles using historical accident probability as external association evidence.

## 3. Methodology

**Figure 1:**
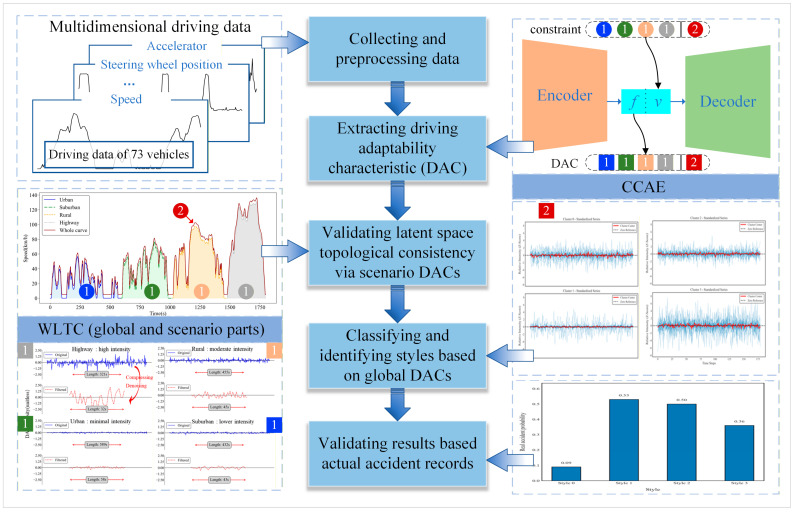
Overall framework of the proposed method.

To address the challenge of contextual entanglement in naturalistic driving data, this section details the proposed unsupervised framework for driving style identification based on context normalization. Specifically, a Constrained Convolutional Autoencoder (CCAE) was developed to extract the Driving Adaptability Characteristic (DAC) from multidimensional CAN-bus data. The autoencoder architecture was selected for its robust feature extraction capabilities from unlabeled data, while the WLTC standard speed profile was introduced as a standardized dynamic reference to guide the construction of comparable latent representations. Figure 1 depicts the overall architecture of the proposed methodology.

Within this framework, multidimensional driving behavior data first undergo necessary preprocessing before being fed into the CCAE model. To examine the cross-sample comparability of the extracted features, scenario-specific DACs—corresponding to the urban, suburban, rural, and highway phases predefined in the WLTC—are then extracted to validate the topological consistency and evolutionary patterns of the latent space. Subsequently, global DAC sequences representing the entire WLTC profile undergo frequency-domain refinement to reduce non-stationary high-frequency noise. These refined global sequences are partitioned using a kernel-mapped clustering algorithm to identify distinct driving styles. Finally, actual vehicle accident records are incorporated as an independent external benchmark to examine whether the identified driving styles are associated with different historical accident probabilities.

### 3.1. Data Collection and Preprocessing

The dataset used in this study was obtained from a commercial vehicle service company and included naturalistic driving records and historical vehicle accident records. The accident records were derived from insurance claims during the preceding policy period and were used only as an external benchmark for evaluating the identified driving styles. The dataset encompassed driving records from 73 real-world vehicles in China, including 31 vehicles with accident records and 42 vehicles without accident records.

The data for each vehicle were collected for approximately two months through CAN-bus signals at a sampling frequency of 1 Hz. Among these signals, variables directly related to driver control inputs, including steering wheel angle, gear position, accelerator pedal position, and brake status, were considered important for modeling driving behavior. In addition, vehicle-state variables that indirectly reflect driving behavior, such as vehicle speed, instantaneous fuel consumption, and engine speed, were incorporated into the model. Table 3 presents representative samples of the data used in this study. Instantaneous fuel consumption denotes the real-time fuel consumption signal, and longitudinal acceleration reflects vehicle longitudinal dynamic response associated with acceleration and braking behavior. Privacy-sensitive information, such as the VIN, was anonymized before analysis. Table 4 displays the vehicle accident records used in this study, where ‘1’ indicates that the vehicle had an accident record during the preceding insurance period and ‘0’ that no accident record was recorded.

The driving behavior data were preprocessed before model training. The main preprocessing steps were as follows:

1. Outlier checking: Because vehicle sensors may record abnormal values under complex operating conditions, basic physical range checks were conducted. For example, vehicle speed was required to fall within 0–61.1 m/s, corresponding to the valid operating range considered in this study.
2. Derived-value checking: Derived variables were calculated from the original CAN-bus signals and then reviewed for abnormal values. For example, segments with abnormal or inconsistent vehicle-speed variation were examined, because such cases may indicate sensor errors, repeated records, or invalid driving phases.
3. Time-based segmentation and normalization: The original continuous data were segmented into time-based driving phases. Segments with insufficient duration were removed because they may not contain enough temporal information for stable feature extraction. After segmentation, the input variables were standardized using Z-score normalization.

### 3.2. Constrained Convolutional Autoencoder (CCAE)

**Figure 2:**
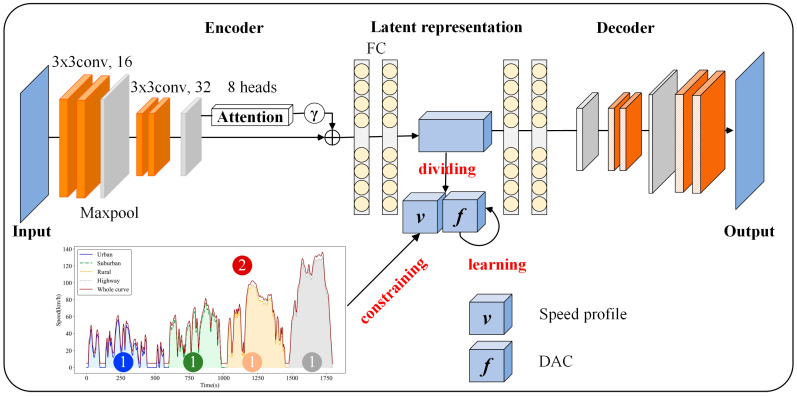
Structure of constrained convolutional autoencoder (CCAE).

To achieve a context-normalized representation learning of personalized driving styles, this study develops a Constrained Convolutional Autoencoder (CCAE) integrated with a global self-attention mechanism. The primary objective of the CCAE is to reduce the influence of heterogeneous naturalistic driving contexts and construct comparable latent representations within a normalized reference space. By introducing a WLTC-guided latent constraint, the model anchors the speed-related latent component to a standardized dynamic reference, allowing the remaining latent representation to capture driver-specific response patterns. Figure 2 illustrates the elaborate architecture of this unsupervised deep learning model developed in this study. The implementation of this framework encompasses four critical components: architecture design, physical topology reconstruction of the latent space and joint loss function optimization, frequency-domain feature refinement, and latent space topological consistency validation.

#### 3.2.1. Architecture Design

The backbone of the CCAE is designed to extract long-term behavioral representations from naturalistic driving sequences. The front-end of the encoder employs multiple convolutional layers coupled with Max-pooling layers. This configuration leverages a local sliding receptive field to capture short-term temporal patterns and reduce the influence of high-frequency fluctuations in the raw CAN-bus signals.

To capture long-range dependencies across driving sequences under complex operating conditions [50], a Single-layer Global Self-attention mechanism is integrated to process the multidimensional joint input sequences. This mechanism computes three matrices—Queries (Q), Keys (K), and Values (V)—through learnable weight matrices $W_{Q}$, $W_{K}$, $W_{V}$ that facilitates the realization of Equations (1)–(3):

(1) $$Q=X_{at}W_{Q}$$

(2) $$K=X_{at}W_{K}$$

(3) $$V=X_{at}W_{V}$$

where $X_{at}$ represents the feature map entering the attention module, and $W_{Q}$, $W_{K}$, $W_{V}$ are the respective learnable weight parameters. The attention weights, which determine the relative importance of each time step across the global time axis, are derived via a Softmax operation:

(4) $$Weight=Softmax\left(\frac{QK^{T}}{\sqrt{D}}\right)$$

where $K^{T}$ is the transposed matrix of *K*, $\sqrt{D}$ serves as a scaling factor to prevent excessively large dot-product values and stabilize the Softmax computation. The output x is then computed as Weight·V. To regulate the contribution of the self-attention mechanism, a learnable factor *γ* is introduced to obtain the final enhanced feature map ${X_{at}}^{\prime }$:

(5) $${X_{at}}^{\prime }=X_{at}+\gamma x$$

The detailed architecture and training configuration of the proposed CCAE are summarized in Table 5. The input sequence length was set to 3600 to provide sufficient temporal information for mapping each sample to the WLTC-aligned latent representation with a length of 1800.

#### 3.2.2. Physical Topology Reconstruction and Joint Loss Function

Following the encoding of the multidimensional joint input sequences, the model constructs a reference-constrained latent representation. The compressed latent variable matrix is defined as $Z\in R^{T\times 2}$, where T corresponds to the physical duration of selected speed profile. The network forcibly partitions Z along the feature channel dimension into two independent subspace sequences:

(6) $$Z=\left[v,f\right]$$

where $v\in R^{T\times 1}$ represents the speed-related latent sequence guided by the reference speed profile, and $f\in R^{T\times 1}$ denotes the residual latent sequence used to characterize driver-specific response patterns.

To implement context-normalized representation learning, a joint loss function is constructed using a reconstruction term and a reference-constraint term. The reconstruction loss (Loss1) preserves the basic signal restoration capability of the autoencoder by minimizing the Mean Squared Error (MSE) between the decoder output and the original multidimensional input:

(7) $$Loss1=\frac{1}{n}\sum_{i=1}^{n}{\left({DA}_{i}^{\prime }-{DA}_{i}\right)}^{2}+\frac{1}{n}\sum_{i=1}^{n}{\left({VS}_{i}^{\prime }-{VS}_{i}\right)}^{2}$$

where n is the input sequence length, DA and ${DA}^{\prime }$ are the original and reconstructed driving operations, such as longitudinal acceleration and steering position, and VS and ${VS}^{\prime }$ are the corresponding original and reconstructed speed sequences. The reference-constraint loss (Loss2) uses a standardized speed profile (sv) to guide the speed-related latent variable (v):

(8) $$Loss2=\frac{1}{n}\sum_{i=1}^{n}{\left(v_{i}-{sv}_{i}\right)}^{2}$$

**Figure 3:**
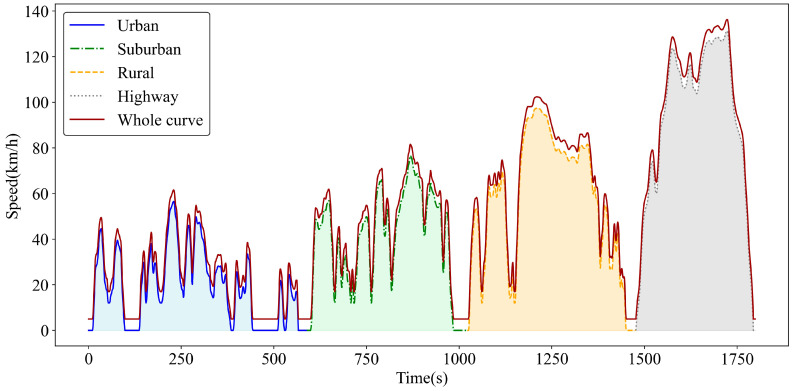
Speed profile of WLTC.

In terms of selecting a suitable standard vehicle speed profile (sv) for this constraint, the Worldwide harmonized Light-duty vehicles Test Cycle (WLTC) was chosen (length n = 1800), as illustrated in Figure 3. WLTC is a widely adopted driving cycle for measuring fuel consumption and emissions of light-duty vehicles in regions including the European Union, China, and Japan. It contains four phases with different speed ranges and dynamic characteristics, corresponding to urban, suburban, rural, and highway driving conditions [51]. Compared with the New European Driving Cycle (NEDC), WLTC contains more diverse speed variations and has been reported to better reflect dynamic driving characteristics [52]. Therefore, the WLTC speed curve is used in this study as a standardized dynamic reference for latent-space alignment and cross-driver comparison.

The total optimization objective is defined as:

(9) Loss=Loss1+Loss2

Under this framework, the decoder restores the multidimensional driving sequences, while the speed-related latent sequence (v) is guided by the WLTC reference profile. Because the speed-related component is explicitly constrained by a common reference trajectory, the residual latent sequence (f) retains the driver-specific response information required for driving style characterization. Accordingly, (f) is defined as the Driving Adaptability Characteristic (DAC). From the perspective of human–vehicle closed-loop control, DAC describes the normalized response pattern exhibited by a driver or vehicle under the same reference speed trajectory. When different drivers are mapped to a common dynamic reference, their differences are reflected in the residual response sequence, including variations in pedal modulation, braking response, steering adjustment, and other control-related behaviors. Therefore, DAC provides a comparable latent representation for subsequent unsupervised driving style identification.

#### 3.2.3. Frequency-Domain Feature Refinement

Following the joint optimization and initial extraction of latent features, cross-segment aggregation and frequency-domain refinement are introduced at the CCAE output to transform segment-level DAC fragments into vehicle-level global driving style representations. For a specific driver’s long-period naturalistic driving data, a fixed time window is applied to segment the continuous data into a sub-dataset containing K fragments. By feeding these fragments individually into the autoencoder guided by the WLTC reference profile, the raw CAN-bus data sequences are mapped and compressed onto a baseline time axis equivalent to the WLTC cycle length of 1800 steps. This process yields K corresponding latent feature sequences, which serve as the preliminary DAC fragments.

For a single driving fragment, the extracted latent sequence $f_{k}(t)$ may still contain segment-specific disturbances caused by temporary traffic interactions or local operating conditions. Therefore, $f_{k}(t)$ can be expressed as the superposition of a driver-specific style component and a segment-specific disturbance term:

(10) $$f_{k}\left(t\right)=f_{style}\left(t\right)+\varepsilon_{k}\left(t\right),\;t\in \left[1,1800\right]$$

where $f_{style}\left(t\right)$ denotes the stable driver-specific response component under the WLTC-based reference, and $\varepsilon_{k}\left(t\right)$ represents segment-specific contextual disturbances, such as sudden congestion, temporary traffic conflicts, or emergency avoidance. By averaging DAC fragments across the segment dimension, these local disturbances are reduced:

(11) $$\bar{f}\left(t\right)=\frac{1}{K}\sum_{k=1}^{K}f_{k}\left(t\right)=f_{style}\left(t\right)+\frac{1}{K}\sum_{k=1}^{K}\varepsilon_{k}\left(t\right)$$

As K increases, the averaged disturbance term is expected to be weakened, allowing $\bar{f}\left(t\right)$ to better represent the global DAC of the driver or vehicle.

The aggregated DAC is further refined using Fast Fourier Transform (FFT)-based low-pass filtering. By retaining the front low-frequency energy bands and reconstructing the signal via Inverse FFT (IFFT), high-frequency operational fluctuations are eliminated, yielding the global steady-state evolution feature f. The global characteristics of f are quantified using Standard Deviation (SD) and Mean Absolute Value (MAV):

(12) $$SD=\sqrt{\frac{1}{N}\sum_{i=1}^{N}{\left(f_{i}-\mu \right)}^{2}}$$

(13) $$MAV=\frac{1}{N}\sum_{i=1}^{N}\left|f_{i}\right|$$

where N is the sequence length (1800) and μ is the mean value.

#### 3.2.4. Latent Space Topological Consistency Validation

In the deployment strategy of the proposed network, a vehicle-specific training approach is adopted, where an individual CCAE model is fitted to the historical dynamics of each driver or vehicle to establish a personalized latent mapping. However, ensuring that these high-level latent features are consistent and comparable across independently trained models is a prerequisite for subsequent unsupervised clustering. To this end, a validation mechanism is developed based on the ordered phase structure of the WLTC speed profile. As shown in Figure 3 and Table 6, the four WLTC phases—Urban, Suburban, Rural, and Highway—provide standardized driving references with different speed levels, acceleration characteristics, and dynamic ranges. Therefore, they can be used as graded reference conditions to examine whether the extracted Scenario DACs exhibit consistent latent-space evolution across independent vehicle-specific models.

Based on this premise, the validation logic for latent space consistency is defined as follows: when the context-normalization mechanism constructs comparable latent representations, the Scenario DACs extracted under the four WLTC phases should exhibit a coherent ordered evolution across the standardized reference conditions. Such consistent evolutionary patterns indicate that the latent representations extracted from independently trained models are aligned in a comparable reference space, providing a basis for cross-individual driving style identification.

To quantitatively evaluate the morphological evolution of Scenario DACs, the SD and MAV are used as compact statistical descriptors. The consistency is verified through a dual-track statistical framework:

- Page’s Trend Test: This test is applied to assess intra-individual trajectory evolution. By treating each driver or vehicle as an independent block, it evaluates whether the latent features consistently follow a monotonic trend across the ordered WLTC phases. A significant result indicates that the Scenario DACs of different individuals exhibit consistent phase-wise evolution under the same reference sequence.
- Jonckheere–Terpstra (JT) Test: This test is used to examine the inter-phase distributional ordering of Scenario DAC statistics. It evaluates whether the numerical distributions of SD and MAV across the four WLTC phases follow an ordered trend. A significant result indicates that the WLTC-based reference constraint helps reduce divergent scale drift among independently trained latent spaces.

The validation results from these two statistical tests support the cross-sample comparability of the extracted DACs and provide a quantitative basis for subsequent unsupervised driving style identification.

### 3.3. Unsupervised Style Clustering Based on Kernel K-Means

Following the extraction of the refined DAC waveforms, an unsupervised clustering framework is constructed to identify distinct driving styles. The extracted DACs characterize driver-specific normalized response patterns, which may contain temporal variations, such as reaction delays, and scale variations, such as differences in response amplitude. Traditional Euclidean K-means is known to be sensitive to local phase shifts, while shape-based clustering algorithms (such as KShape) typically rely on intra-sample normalization and may weaken amplitude-related information. Because both temporal alignment and response magnitude are relevant for driving style characterization, a kernel-based clustering strategy is adopted in this study.

To accommodate potential temporal elasticity while preserving the magnitude of driving responses, this study employs Kernel K-means combined with the Global Alignment Kernel (GAK). By implicitly mapping the features into a high-dimensional Reproducing Kernel Hilbert Space (RKHS), the squared distance between a sample $x_{i}$ and a cluster centroid $\mu_{c}$ is computed using the Kernel Trick:

(14) $$D^{2}\left(x_{i},\mu_{c}\right)=K\left(x_{i},x_{i}\right)-\frac{2}{\left|C_{c}\right|}\sum_{x_{j}\in C_{c}}K\left(x_{i},x_{j}\right)+\frac{1}{{\left|C_{c}\right|}^{2}}\sum_{x_{j},x_{l}\in C_{c}}K\left(x_{j},x_{l}\right)$$

where $C_{c}$ represents the set of samples assigned to cluster c, and $\left|C_{c}\right|$ denotes the cluster size.

As a positive definite variant of Dynamic Time Warping (DTW), GAK incorporates an exponential soft-minimum mechanism to handle time series alignments. For two equal-length sequences x and y, the GAK is defined as:

(15) $$K_{GAK}\left(x,y\right)=\sum_{\pi \in A\left(x,y\right)}\prod_{i=1}^{\left|\pi \right|}k\left(x_{\pi_{1}\left(i\right)},y_{\pi_{2}\left(i\right)}\right)$$

where $A\left(x,y\right)$ denotes the set of all valid monotonic alignment paths, and k(·,·) is the local kernel function measuring point-wise deviations. This kernel alignment provides a flexible and robust metric for measuring sequence similarity in naturalistic driving data. Finally, the optimal number of clusters is determined using the Kernel Silhouette Score, which evaluates cluster cohesion and separation within the nonlinear space. A higher Kernel Silhouette Score indicates clearer within-cluster similarity and between-cluster separation and is therefore used as the main criterion for selecting the number of driving style clusters.

## 4. Results Analysis

Following the data collection and preprocessing procedures detailed in Section 3.1, a final dataset comprising approximately 3.65 million valid time series entries (sampled at 1 Hz) was retained for further analysis. This dataset included 73 independent vehicles, among which 31 vehicles had historical accident records during the preceding policy period and 42 vehicles had no recorded accidents. The descriptive statistics of the core features are presented in Table 7.

The proposed CCAE model was implemented in Python 3.9 using the PyTorch 2.2.2 framework and trained on a server equipped with dual NVIDIA RTX 3090Ti GPUs. As summarized in Table 5, each input sample was set to a length of 3600, which is longer than the WLTC-aligned latent representation length of 1800. For each vehicle, a vehicle-specific training strategy was adopted, and the sequence data were randomly partitioned into training and test sets at a ratio of 80% and 20%, respectively. All input variables were standardized using Z-score normalization before model training.

During optimization, the global maximum training epochs were set to 300, with a batch size of 8. The network was optimized using the Adam optimizer with an initial learning rate of 0.001. A weight decay coefficient of 0.0001 was introduced for regularization. These settings were used to stabilize the training process and reduce potential overfitting during latent representation learning.

### 4.1. Performance of CCAE

**Figure 4:**
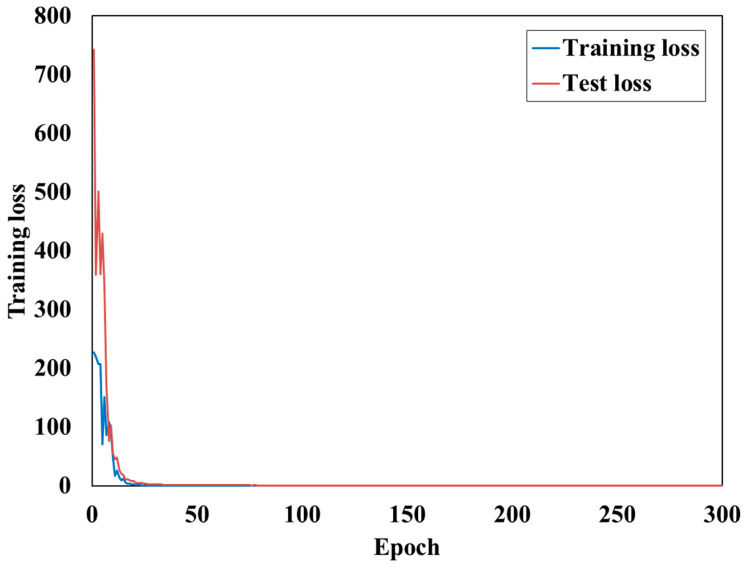
Loss value change against epochs.

The primary task of the CCAE is to achieve accurate reconstruction of multidimensional driving states while learning WLTC-guided latent representations. Figure 4 illustrates the convergence of the joint loss function during the iterative training process.

During the initial phase of training, the model undergoes rapid exploration of the feature space, resulting in relatively high loss values for both the training and test sets. As the number of epochs increases, the network progressively captures the underlying topological relationships within the data, leading to a steady and significant decline in the joint loss. Ultimately, the model achieves stable convergence, with the training loss reaching approximately 0.03 and the test loss stabilizing at 0.61. Throughout the training cycle, the test loss curve remains close to the training loss, indicating that the architecture maintains stable reconstruction performance without obvious overfitting under the vehicle-specific training setting.

**Figure 5:**
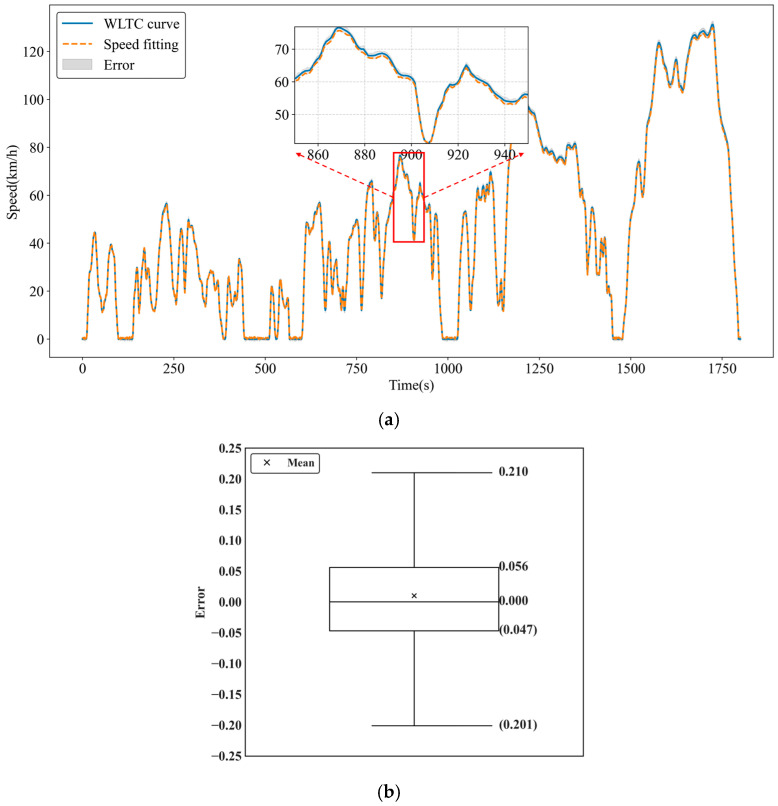
Speed fitting performance: (**a**) speed error presentation. (**b**) overall speed error statistic.

As established in Section 3.2, the CCAE introduces a WLTC-guided reference constraint to align the speed-related latent sequence (v) with the standardized WLTC speed profile. To evaluate the effectiveness of this reference constraint across the sample set, Figure 5 displays the speed tracking performance for a randomly selected sample, together with the statistical distribution of fitting errors for all 73 vehicles.

The results demonstrate that the reconstructed speed profiles exhibit good agreement with the WLTC reference profile, capturing major acceleration and deceleration variations along the standardized speed trajectory. The box plot of the error distribution shows that the majority of speed errors are confined within the ([−1.0, 1.0]) km/h interval. This level of tracking precision satisfies the reference accuracy requirement considered in this study [53]. These findings indicate that the proposed WLTC-guided reference constraint effectively aligns the speed-related latent sequence across the 73 vehicle-specific models, providing a standardized basis for subsequent DAC extraction and cross-driver comparison.

### 4.2. Latent Space Topological Consistency Validation Results

Based on the verified reconstruction performance and WLTC-guided reference alignment of the CCAE, this section investigates the topological consistency of the extracted latent representations. For each driver in the dataset, scenario-specific waveforms, namely Scenario DACs, were extracted using speed fragments from the four WLTC phases: Urban, Suburban, Rural, and Highway.

**Figure 6:**
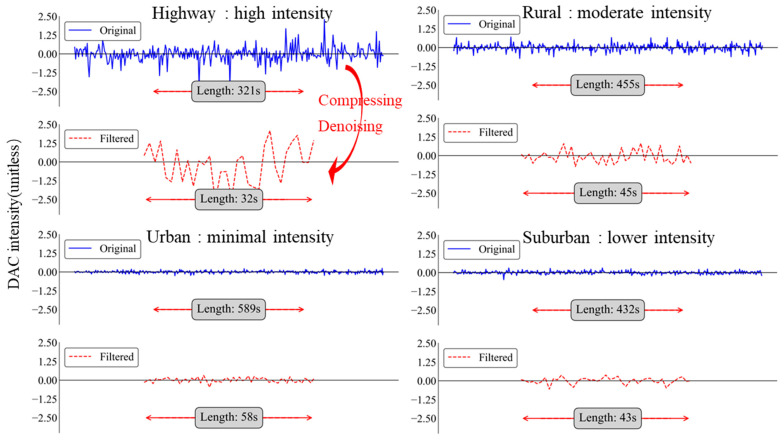
Four scenario DACs of random sample: original vs. low-pass filtered.

To highlight low-frequency response trends in the Scenario DACs, the FFT-based low-pass filtering mechanism described in Section 3.2.3 was applied to the raw DAC sequences. The retention frequency band was set to the top 10%, and the sensitivity of this truncation ratio is further examined in Section 5.2. This operation aims to reduce high-frequency non-stationary disturbances and retain the main low-frequency evolution pattern of the DAC sequences. Figure 6 illustrates a representative comparison between the original and filtered waveforms for a randomly selected driver across the four WLTC phases. The results indicate that the filtering process reduces dense high-frequency fluctuations, while the reconstructed waveforms preserve the main low-frequency response structure. This refinement provides a more stable input for subsequent statistical validation and clustering.

The four WLTC phases provide standardized reference conditions with different speed levels, acceleration characteristics, and dynamic ranges. Therefore, if the proposed context-normalization mechanism produces comparable latent representations across independently trained vehicle-specific models, the Scenario DAC statistics should exhibit a coherent ordered evolution across these phases. Table 8 summarizes the SD and MAV values of the Scenario DACs across the four phases for the entire sample set. The results show a clear monotonic trend from Urban to Highway: the global mean SD increases from 1.11 in the Urban phase to 2.88 in the Highway phase, while the mean MAV rises from 0.89 to 2.37. This ordered evolution indicates that the extracted Scenario DACs respond consistently to the standardized WLTC phase sequence.

To assess the comparability of the latent features across independent vehicle models, a dual-track statistical trend validation framework was implemented, as presented in Table 9. Page’s Trend Test was applied to evaluate the intra-individual trajectory evolution by treating each driver as an independent block. The results (MAV: P = 1.13 × 10^−6^; SD: P = 4.44 × 10^−7^) significantly rejected the null hypothesis, indicating that the Scenario DAC statistics follow a consistent monotonic trend across the ordered WLTC phases. Jonckheere–Terpstra (JT) Test was used to assess the inter-phase distributional ordering. The results (MAV: P = 3.67 × 10^−4^; SD: P = 2.56 × 10^−4^) further support the ordered distributional differences among the four phases. Together, these results indicate that the WLTC-guided reference constraint helps maintain comparable latent-scale evolution across independently trained models.

These dual statistical validations support the cross-sample comparability of the extracted DACs. By demonstrating consistent intra-individual evolution and ordered inter-phase distributional differences, this section provides a quantitative basis for subsequent unsupervised driving style identification.

### 4.3. Unsupervised Driving Style Clustering Based on Kernel K-Means

Prior to global clustering, cross-sectional standardization was applied along the time dimension across all samples. This operation reduced dimensional-scale discrepancies while preserving between-sample amplitude differences in the DAC sequences. Based on the refined global DAC sequences, the Kernel K-means algorithm equipped with the Global Alignment Kernel (GAK) was deployed to construct the unsupervised clustering framework.

In the kernel mapping process, the scale parameter σ dictates the sensitivity of the kernel matrix to feature discrepancies. To reduce the influence of arbitrary parameter selection, a data-driven heuristic baseline combined with a multiplier grid search strategy was adopted. The multiplier was evaluated within the [0.1, 2.0] interval to optimize the cluster structure in the kernel space. Through this evaluation, the optimal multiplier was established at 0.2 (i.e., σ = 0.2 × Base σ). This configuration provided sufficient nonlinear resolution for distinguishing DAC sequence patterns while avoiding trivial solutions caused by over-smoothing or excessive fragmentation.

**Figure 7:**
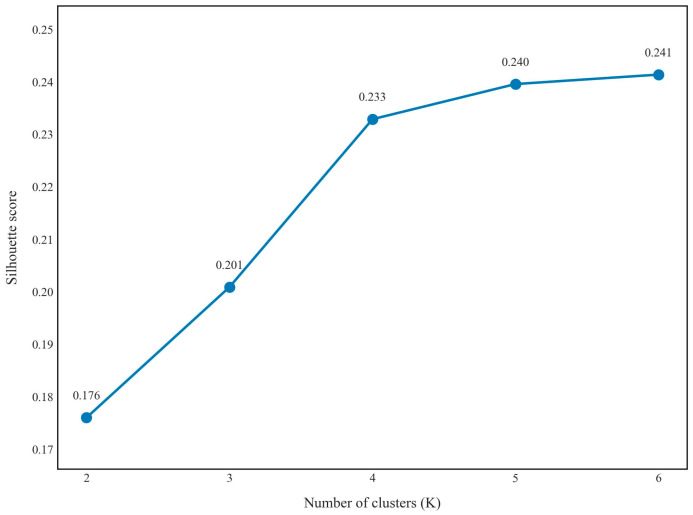
Silhouette Score curve across different cluster numbers.

With the optimal scale parameter determined, the Kernel Silhouette Score was utilized to quantify the optimal number of clusters K. As illustrated in Figure 7, the silhouette score exhibits a steep ascent as K increases from 2 to 4, reaching a distinct peak of 0.233 at K = 4. Although the score showed a slight increase at larger (K) values, such as 0.241 at (K = 6), the marginal improvement was limited and would lead to finer cluster partitions with reduced interpretability under the current vehicle-level sample size. Therefore, considering both kernel-space separability and practical driving style interpretation, K = 4 was selected as the global cluster number.

**Figure 8:**
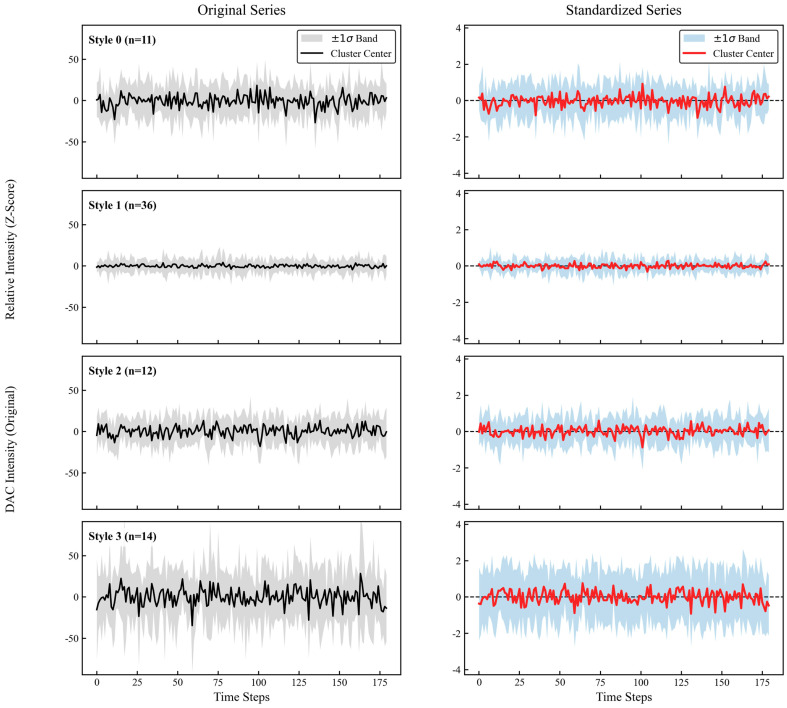
Visualization of DAC sequences for the four driving styles.

Consequently, the entire sample was partitioned into four distinct driving style clusters. The central tendencies and statistical dispersion (±1σ shaded bands) of their corresponding global DAC sequences are visualized in Figure 8. To summarize the response morphology of each cluster, the average Standard Deviation (SD) and Mean Absolute Value (MAV) were calculated, as shown in Table 10. It should be noted that SD and MAV describe the variability and magnitude of the normalized DAC response under the WLTC reference, and should not be directly interpreted as raw driving intensity or safety risk.

Style 1 exhibits the lowest DAC fluctuation level, with SD = 4.43 and MAV = 3.60, indicating a low-variation normalized response pattern under the WLTC reference. Style 2 presents moderate-low metrics, with SD = 19.33 and MAV = 15.53, suggesting a higher but still relatively constrained response variability compared with Style 1. Style 0 shows intermediate values, with SD = 22.45 and MAV = 18.16, representing a moderate normalized response pattern. Style 3 demonstrates the highest statistical values, with SD = 32.54 and MAV = 25.92, indicating a high-variation normalized response pattern.

To further examine whether the identified driving styles correspond to distinguishable vehicle operating characteristics, the original CAN-bus signals were traced back and summarized at the vehicle level, as shown in Table 10. This backtracking analysis was used for post-hoc interpretation and was not involved in DAC extraction or clustering. The results show that the four driving styles exhibit clear differences in raw operating features, including speed level, speed variability, engine speed, instantaneous fuel consumption, and steering variability.

Specifically, Style 1 shows the lowest DAC fluctuation under the WLTC reference, but it has the highest mean speed, speed variability, engine speed, and instantaneous fuel consumption in the original CAN-bus data. Its mean speed reaches 15.56 m/s, corresponding to approximately 56 km/h, whereas the other three styles show much lower mean speeds ranging from 1.43 to 2.59 m/s. This indicates that the DAC-derived styles are associated with markedly different real-world operating profiles. Meanwhile, the low DAC fluctuation of Style 1 suggests that DAC magnitude does not simply duplicate raw speed level or raw operating intensity but characterizes a normalized response pattern under the standardized reference trajectory.

Style 2 has a relatively low mean speed but higher engine speed and instantaneous fuel consumption than Styles 0 and 3, suggesting that the identified styles are not determined solely by speed level. Styles 0 and 3 both show relatively low mean speed and engine operating intensity, but they differ substantially in DAC fluctuation, indicating that the DAC representation captures response-pattern differences beyond conventional vehicle-level statistics.

Overall, the four identified styles exhibit distinguishable DAC waveform morphologies under the standardized WLTC reference. These differences provide a basis for interpreting driving styles in the normalized latent space. Furthermore, the raw CAN-bus feature backtracking results in Table 10 show that the DAC-derived styles also correspond to distinguishable real-world operating characteristics, including speed level, speed variability, engine speed, instantaneous fuel consumption, and steering variability. These findings indicate that the proposed DAC representation does not merely generate mathematical partitions in the latent space but captures driving style groups with observable physical differences in the original CAN-bus data. The relationship between the identified styles and historical accident probabilities is examined separately in the following section.

### 4.4. Result Validation Using Historical Accident Records

**Figure 9:**
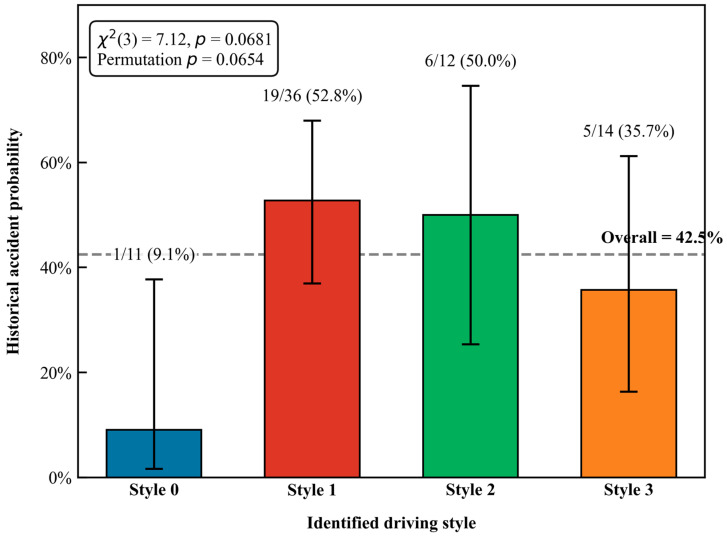
Comparison of historical accident probabilities across driving styles.

To examine whether the identified data-driven driving styles are associated with real-world historical accident records, historical vehicle accident records were introduced as an independent external benchmark. Figure 9 illustrates the historical accident probabilities of the four identified driving styles. The bars represent vehicle-level accident probabilities, the error bars indicate 95% Wilson confidence intervals, and the dashed horizontal line denotes the overall accident probability of the full sample.

The results show large observed differences in historical accident probability among the four styles. Style 1 has the highest observed accident probability, with 19 accident-record vehicles among 36 vehicles (52.8%), followed by Style 2 with 6 out of 12 vehicles (50.0%) and Style 3 with 5 out of 14 vehicles (35.7%). In contrast, Style 0 has the lowest observed accident probability, with 1 out of 11 vehicles (9.1%). The highest observed accident probability is approximately 5.8 times that of the lowest group in this dataset. These results indicate that the identified driving styles are associated with different historical accident probabilities.

To further evaluate the statistical evidence for this association, a chi-square test of independence was conducted on the accident frequency distribution across the four styles. The global test statistic was 7.12, with a corresponding *p*-value of 0.0681. The expected-frequency check showed that only 12.5% of the expected cell counts were below 5 and none were below 1, generally satisfying the commonly used assumption for the chi-square test. Considering the limited vehicle-level sample size (N = 73), a 2000-iteration random permutation test was also conducted, yielding a global *p*-value of 0.0654. Both tests provide consistent but marginal evidence of association between the identified driving styles and historical accident records. Fisher’s exact tests were also used to examine differences in historical accident probabilities among the identified styles. When Style 0 was compared with the remaining three styles combined, a lower historical accident probability was observed for Style 0 (two-sided Fisher’s exact test, *p*-value = 0.019). Among the individual pairwise comparisons, the largest difference was observed between Style 0 and Style 1 (*p*-value = 0.014), while the remaining individual pairwise comparisons did not show clear statistical separation in the current sample. Together, these results indicate an observed association between the identified driving styles and historical accident probabilities. Within this overall pattern, Style 0 exhibits the most evident difference from the other styles in the current sample, whereas the numerical differences among Styles 1–3 require further verification using larger independent datasets.

Combined with the DAC morphology and raw CAN-bus feature backtracking results in Section 4.3, the accident-record analysis provides further insight into the external relevance of the identified styles. Style 1 shows the lowest DAC fluctuation under the WLTC reference, but its original CAN-bus statistics indicate a substantially higher operating speed level, speed variability, engine speed, and instantaneous fuel consumption than the other styles. Its higher historical accident probability may therefore be related to a real-world operating profile characterized by higher speed and engine load, although this relationship should be interpreted as an observed association rather than a causal mechanism. Style 2 also shows a relatively high accident probability, despite its lower mean speed, and its higher engine speed and instantaneous fuel consumption compared with Styles 0 and 3 suggest that the accident-probability differences are not determined solely by speed level. In contrast, Style 3 has the largest DAC fluctuation but a lower accident probability than Styles 1 and 2, indicating that DAC magnitude alone should not be directly interpreted as accident risk. Style 0 combines relatively moderate DAC morphology with lower raw operating intensity and shows the lowest observed accident probability in this dataset.

These results suggest that the identified driving styles are associated with different historical accident probabilities, and that this association is better understood by jointly considering normalized DAC response patterns and raw CAN-bus operating characteristics. Importantly, the accident records were not used in DAC extraction or clustering and thus provide independent external supporting evidence for the practical relevance of the proposed driving style identification framework. However, the results should not be interpreted as causal evidence or exposure-adjusted accident risk estimates, because actual traffic accidents may also be affected by unobserved factors such as driving exposure, road environment, weather, traffic conditions, and accident responsibility.

### 4.5. Ablation Analysis of the WLTC-Guided Reference Constraint

To further examine the contribution of the WLTC-guided reference constraint, an ablation experiment was conducted by removing the reference-constraint loss (Loss2) from the CCAE. In this ablated model, the network was optimized only using the reconstruction loss (Loss1), while all other settings, including the network architecture, input sequence length, preprocessing procedure, FFT-based refinement, and Kernel K-means clustering strategy, were kept unchanged. For a controlled comparison, the number of clusters was fixed at (*K* = 4), consistent with the proposed framework.

The comparison results are summarized in Table 11. Without the WLTC-guided reference constraint, the Kernel Silhouette Score decreased substantially from 0.233 to 0.0194, indicating that the latent representations learned by the reconstruction-only autoencoder had much weaker cluster separability in the kernel space. In addition, the cluster sizes changed to 13, 15, 33, and 12, and the corresponding historical accident probabilities were 0.4615, 0.4000, 0.4242, and 0.4167, respectively. These probabilities were close to the overall accident probability of the full sample, suggesting that the ablated representations provided limited external differentiation with respect to historical accident records.

Compared with the reconstruction-only autoencoder, the proposed CCAE produced more distinguishable DAC-based clusters in both kernel-space separability and external accident-record association. Specifically, the MD value increased from 0.0131 to 0.1265 after introducing Loss2, indicating that the proposed reference constraint enhanced the cluster-level differentiation of historical accident probabilities. More importantly, this improvement was achieved without using accident labels during representation learning or clustering.

These results support the necessity of the WLTC-guided reference constraint in the proposed framework. By anchoring the speed-related latent sequence to a standardized dynamic reference, Loss2 helps reduce unconstrained latent-space drift and guides the model to extract more comparable residual response patterns, namely DACs. Therefore, the ablation analysis further supports that the proposed CCAE is not merely a reconstruction-based dimensionality reduction model; instead, the WLTC-guided constraint plays an important role in constructing effective latent representations for unsupervised driving style identification.

## 5. Discussion and Effectiveness Analysis

The preceding sections showed that the proposed framework can identify driving styles with distinguishable DAC morphologies, raw CAN-bus operating characteristics, and different historical accident probabilities. To further evaluate the comparative performance and robustness of the proposed methodology, this section conducts a comparison with representative feature extraction models and a sensitivity analysis of the frequency-domain refinement mechanism.

### 5.1. Comparison of Different Feature Extraction Models

To compare different feature extraction models under a common external reference, the Mean Deviation (MD) of historical accident probabilities was introduced as a descriptive evaluation metric:

(16) $$MD=\frac{\sum_{k=1}^{K}V_{k}\left|P_{k}-\bar{P}\right|}{\sum_{k=1}^{K}V_{k}}$$

where K is the total number of clusters (K = 4), $V_{k}$ denotes the sample size of the k-th cluster, V is the total number of samples, $P_{k}$ represents the historical accident probability of the k-th cluster, and P is the global average accident probability across all samples. A higher MD value indicates that the cluster-level accident probabilities deviate more clearly from the overall average, suggesting stronger external differentiation with respect to historical accident records.

To evaluate the comparative performance of the proposed CCAE, five representative baseline models were selected for parallel experimentation under the same input data and preprocessing settings:

- Traditional Feature Engineering (Traditional): A parametric evaluation model reliant on domain expert knowledge, utilizing low-dimensional statistical features such as annualized mileage, mean instantaneous fuel consumption, and speed standard deviation.
- FCAE: A Fully Connected Autoencoder without any physical constraints.
- DBN: A Deep Belief Network representing unconstrained deep feature extraction.
- FCAEN: A Fully Connected Autoencoder integrated with the WLTC physical constraint.
- CRAEN: An autoencoder combining Convolutional Neural Networks (CNN) and Recurrent Neural Networks (RNN), integrated with the WLTC physical constraint.

To ensure that the clustering strategy matched the form of each model output, different clustering algorithms were adopted according to the representation type. Unconstrained models, including Traditional, FCAE, and DBN, produced static low-dimensional vectors and were clustered using standard K-means in Euclidean space. Models incorporating the WLTC-guided constraint, including FCAEN, CRAEN, and CCAE, produced dynamic latent sequences and were clustered using Kernel K-means equipped with GAK after cross-sectional standardization.

**Figure 10:**
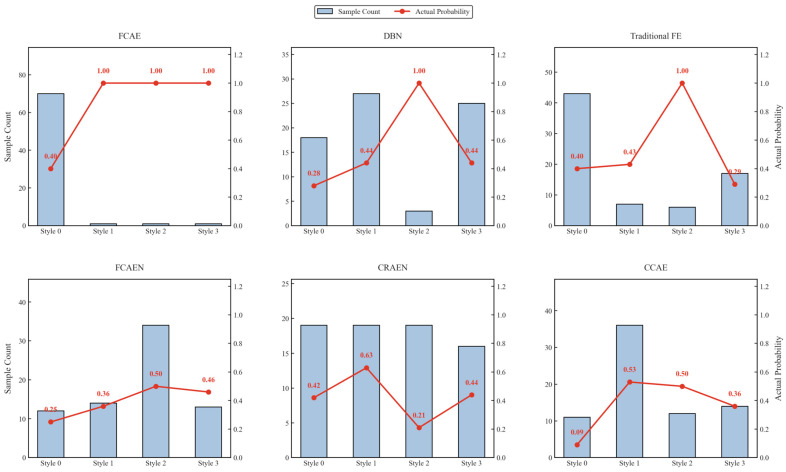
Cluster distribution and historical accident probability across different feature extraction models.

**Figure 11:**
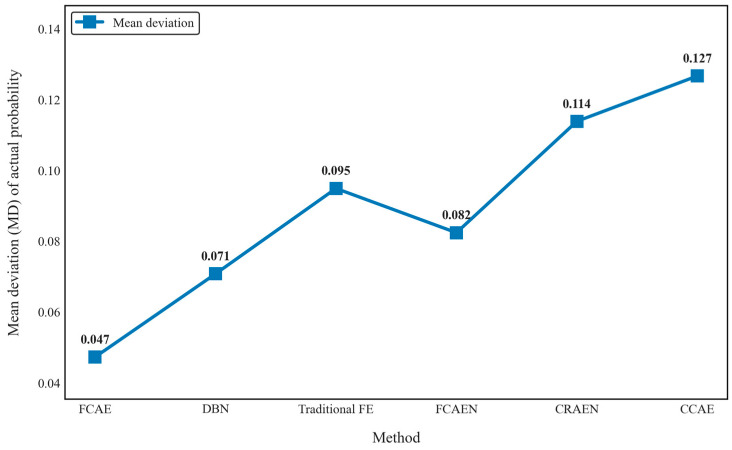
Mean deviation of historical accident probabilities across different feature extraction models.

Figure 10 and Figure 11 present the cluster distributions and MD values obtained from the different models. Several observations can be drawn from the results.

First, the WLTC-guided reference constraint contributes to improving the external differentiation of the learned representations. Among the unconstrained deep models, FCAE and DBN obtained relatively low MD values of 0.047 and 0.071, respectively. This suggests that when no common reference trajectory is introduced, latent representations learned only from reconstruction objectives may be more affected by heterogeneous driving contexts and may provide limited cluster-level differentiation with respect to historical accident records. After introducing the WLTC-guided reference constraint into the fully connected autoencoder, the MD value increased to 0.082. This improvement indicates that reference-guided latent alignment helps construct more comparable representations across heterogeneous naturalistic driving data.

Second, the Traditional feature engineering approach achieved an MD value of 0.095, indicating that hand-crafted statistical features can provide a certain level of external differentiation. However, these low-dimensional statistical indicators mainly summarize global operating characteristics and may not fully capture the sequential evolution of driving behavior. Therefore, their ability to represent long-term dynamic response patterns remains limited.

Third, models with higher-order temporal representation capability showed improved performance. The CRAEN model, which combines CNN and RNN structures with the WLTC-guided reference constraint, achieved an MD value of 0.114. This result suggests that modeling temporal dynamics is beneficial for extracting driving style representations. However, for long reference sequences such as the 1800-step WLTC profile, recurrent structures may still be limited in capturing global dependencies across the entire sequence.

In comparison, the proposed CCAE achieved the highest MD value of 0.127. By integrating convolutional feature extraction with a global self-attention mechanism, the model can capture both local temporal patterns and long-range dependencies in the WLTC-aligned latent space. As shown in Figure 10, the proposed CCAE produced clusters with more distinguishable historical accident probabilities, including groups with relatively high observed accident probabilities of 0.53 and 0.50, as well as a group with the lowest observed accident probability of 0.09. These results support the comparative advantage of the proposed CCAE in extracting DAC representations for unsupervised driving style identification.

Overall, the comparative results indicate that the proposed framework provides stronger cluster-level differentiation than traditional feature engineering and unconstrained deep representation models under the current dataset. This improvement is attributed to the combination of WLTC-guided reference alignment, convolutional temporal feature extraction, and global self-attention. Nevertheless, the MD-based comparison should be interpreted as an external association analysis based on historical accident records, not as causal evidence of accident-risk prediction.

### 5.2. Sensitivity Analysis of Frequency-Domain Truncation Ratio

In the feature refinement stage, an FFT-based low-pass filtering mechanism was introduced to reduce high-frequency non-stationary fluctuations in the extracted DAC sequences. The baseline truncation ratio was set to 10%, corresponding to a retained sequence dimension of 180. To examine whether the identified driving styles were sensitive to this frequency-domain hyperparameter, a sensitivity analysis was conducted by varying the truncation ratio from 5% to 30%. In addition to the Mean Deviation (MD) of historical accident probabilities, the Adjusted Rand Index (ARI) and Normalized Mutual Information (NMI) were introduced to compare the clustering labels under different truncation ratios with the 10% baseline. These two indicators evaluate the stability of the clustering structure independently of accident labels.

**Figure 12:**
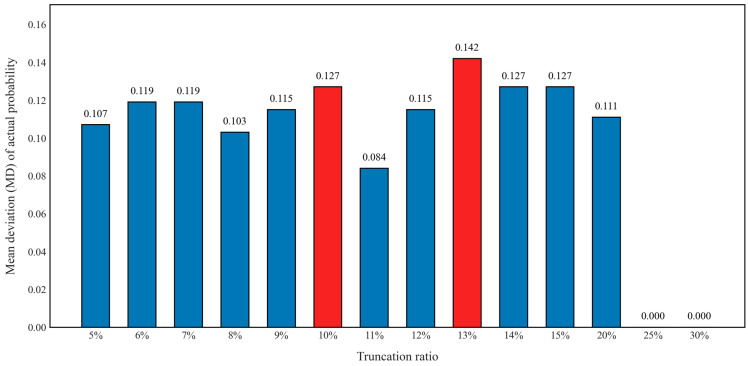
Variation of historical accident probability MD under different FFT truncation ratios.

Table 12 and Figure 12 present the clustering results under different FFT truncation ratios. Overall, the results show that the clustering structure remains largely stable around the 10% baseline. For truncation ratios from 5% to 10%, the ARI values increase from 0.798 to 1.0, and the NMI values increase from 0.807 to 1.0, indicating that the cluster assignments gradually converge to the baseline solution as more low-frequency components are retained. Similar clustering structures are also observed at 12–15%, where the ARI and NMI values remain very high. In particular, the clustering results at 14% and 15% are identical to the 10% baseline.

A local fluctuation is observed at the 11% truncation ratio, where ARI and NMI decrease to 0.525 and 0.386, respectively. This result indicates a local reallocation of boundary samples in the kernel clustering space. However, the clustering structure returns to a highly consistent solution at 12% and 13% and becomes identical to the 10% baseline at 14% and 15%. Therefore, the 11% result is better interpreted as a local boundary-sensitive case rather than a systematic instability of the frequency-domain refinement process.

At higher truncation ratios, the benefit of low-pass filtering becomes more evident. When the truncation ratio increases to 25% and 30%, all samples are merged into a single cluster, and the MD value decreases to 0. This collapse suggests that retaining excessive high-frequency components weakens the distinguishable low-frequency DAC structure required for clustering. Therefore, frequency-domain refinement is important for reducing local high-frequency fluctuations and preserving stable global response patterns in DAC sequences.

The 10% truncation ratio was adopted as the baseline setting according to common low-frequency retention practice in frequency-domain signal refinement. The sensitivity analysis further shows that the results remain stable within the neighboring low-frequency retention range. In particular, the 13% truncation ratio achieved the highest MD value of 0.142, and its clustering structure remained highly consistent with the 10% baseline, as indicated by the high ARI and NMI values. This result suggests that the accident-probability differentiation observed under the baseline setting is not limited to a single truncation ratio but remains reproducible across nearby frequency-domain configurations. Therefore, the sensitivity analysis supports both the rationality of the 10% baseline setting and the robustness of the DAC-based clustering results.

Overall, the sensitivity analysis supports the robustness of the proposed frequency-domain refinement strategy. The DAC-based clustering results remain highly consistent across most truncation ratios around the 10% baseline, and the identified styles maintain distinguishable historical accident probability distributions. These findings indicate that the proposed method is not dependent on a single arbitrary truncation setting, while future studies with larger datasets are still needed to further stabilize statistical inference regarding accident-record associations.

## 6. Conclusions

This study developed an unsupervised framework for identifying driving styles from naturalistic driving data through context-normalized representation learning. Historical accident records were introduced as an independent external benchmark to examine the practical relevance of the identified styles. The main conclusions are as follows:

- A context-normalized CCAE framework was developed to extract WLTC-anchored DAC representations from unlabeled naturalistic CAN-bus data, enabling comparable driving style characterization across heterogeneous driving contexts.
- Four physically interpretable driving styles were identified, showing distinguishable DAC waveform morphologies and raw CAN-bus operating characteristics such as speed level, engine speed, fuel consumption, and steering variability.
- Historical accident records provided external supporting evidence for the practical relevance of the identified styles. The highest observed accident probability was approximately 5.8 times that of the lowest group, and the comparative, ablation, and sensitivity analyses further supported the robustness of the proposed framework.

Overall, the proposed framework provides an interpretable and scalable approach for identifying driving styles from unlabeled naturalistic CAN-bus data. The accident-record analysis provides independent external supporting evidence for the practical relevance of the identified styles. However, the results should not be interpreted as causal evidence or exposure-adjusted accident risk estimates, because accident occurrence may also be affected by driving exposure, road environment, weather, traffic conditions, and accident responsibility. Future studies will use larger-scale datasets with richer contextual and exposure information, such as validated mileage, road type, weather, traffic conditions, maintenance records, and accident severity, to further examine the generalizability of the proposed framework and its applicability in personalized intelligent transportation services.

## Figures and Tables

**Table 1:** Driving data and acquisition means.

Acquisition Means	Data Category	Reference
Human body sensor	Breathe, EEG, ECG, myoelectricity	[9,10,11]
Camera and radar	Face, posture, track, environment	[13,14,15]
CAN-BUS sensor	Speed, accelerator, brake, gear, steering	[16,17,18,19]

**Table 2:** Driving behavior analysis methods.

Type	Approach	Expertise Required	Labelled Data Required
Theoretical modelling	Parametric modelling, simulation modelling	Yes	No
Traditional machine learning	PCA, HMM, Nonparametric Bayes	Yes	No
Supervised deep learning	MLP, CNN, RNN, FC, LSTM	No	Yes
Unsupervised deep learning	RBM, DBN, autoencoder	No	No

**Table 3:** Sample data for driving behavior feature extraction model training.

VIN	Record Timestamp	Instant Consume (L/100 km)	Longitudinal Acceleration (m/s^2^)	Steering Position (Degree)	Vehicle Speed (m/s)	Engine Speed (RPM)
LLA0032xx	2012/1/1 2:02:14	20	0.26	2.5	2.85	1130
LLA0032xx	2012/1/1 2:02:15	20	−0.14	2.5	2.925	1136.75
LLA0032xx	2012/1/1 2:02:16	0	−2	2.5	1.65	1184.75
LLA0032xx	2012/1/1 2:02:17	20	−0.34	2.5	3.525	1200.25

**Table 4:** Samples of vehicle accident records.

VIN	Accident Record
LLA0032xx	0
LLN0028xx	0
LLA0018xx	1
LLN0024xx	1

**Table 5:** Key architecture and training settings of the proposed CCAE.

Item	Configuration	Description
Input sequence	Sequence length = 3600; input dimension = 6	Multidimensional CAN-bus sequence after Z-score normalization
Encoder	Two convolutional layers with 16 and 32 channels; kernel size = (3 × 3); ReLU activation; max-pooling layers	Extracts temporal-feature representations from naturalistic driving sequences
Self-attention module	Multi-head attention; number of heads = 8; embedding dimension = 32; dropout = 0.4	Captures long-range dependencies in the encoded feature sequence
Latent representation	Linear layers: (32 × 600 → 512 → 3600); reshaped as $\left(Z\in R^{1800\times 2}\right)$	The latent matrix is partitioned into $\left(v\in R^{1800\times 1}\right)$ and $\left(f\in R^{1800\times 1}\right)$, where (*f*) is defined as the DAC
Decoder	Symmetric fully connected, unpooling, and transposed convolutional layers	Reconstructs the multidimensional driving sequence
Optimization	Adam optimizer; learning rate = 0.001; weight decay = 0.0001; batch size = 8; maximum epochs = 300	Model training configuration
Data split	80% training set and 20% test set for each vehicle	Vehicle-specific training and testing
Model scale	Approximately 23.38 million trainable parameters	Total trainable parameters of the CCAE

**Table 6:** Comparisons of four parts in WLTC.

	Duration (s)	Average Speed (m/s)	Maximum Speed (m/s)	Minimum Acceleration (m/s^2^)	Maximum Acceleration (m/s^2^)
Urban	589	5.25	15.69	−1.5	1.611
Suburban	433	10.94	21.27	−1.5	1.611
Rural	455	15.69	27.06	−1.5	1.666
Highway	323	25.47	36.47	−1.44	1.055

**Table 7:** Descriptive statistics for the subset.

	Longitudinal Acceleration (m/s^2^)	Vehicle Speed (m/s)	Engine Speed (RPM)	Steering Position (Degree)	Instant Consume (L/100 km)
Mean	−0.23	11.64	793.27	3.77	4.63
Std	0.49	17.01	608.56	115.73	6.79
Min	−10.	0	0	−544.5	0
25%	−0.34	0	0	−6.0	0
50%	−0.24	0.075	771	1.5	0
75%	−0.12	20.4	1229.25	8.5	8.0
Max	8.8	60	6185.5	540	51.1

**Table 8:** SD and MAV statistic of scenario DACs for all samples after low-pass filtering.

	Urban (All)	Suburban (All)	Rural (All)	Highway (All)
SD	1.11	1.53	1.63	2.88
MAV	0.89	1.24	1.30	2.37

**Table 9:** Statistical results of trend tests.

Test Method	Statistics (MAV)	*p* Value (MAV)	Statistics (SD)	*p* Value (SD)
Page’s Trend Test	851.0	1.13e × 10^−6^ *	854.0	4.44 × 10^−7^ *
Jonckheere–Terpstra	3639.0	3.67e × 10^−4^ *	3661.0	2.56 × 10^−4^ *

Note: * Indicating a significant correlation at the *p* < 0.05 level.

**Table 10:** Vehicle-level raw CAN-bus feature backtracking statistics of the identified driving styles.

Style	Count	DAC SD	DAC MAV	Mean Speed (m/s)	Speed Std. (m/s)	Mean Absolute Longitudinal Acceleration (m/s^2^)	Engine Speed	Instant Fuel Consumption	Steering Std.
Style 0	11	22.45	18.16	1.55	5.44	0.58	340.11	1.75	127.36
Style 1	36	4.43	3.60	15.56	21.37	0.75	843.88	4.41	101.41
Style 2	12	19.33	15.53	2.59	6.43	0.49	459.27	2.65	169.35
Style 3	14	32.54	25.92	1.43	5.75	0.71	293.60	1.49	139.46

**Table 11:** Ablation analysis results of the WLTC-guided reference constraint.

Model Setting	Loss Function	Kernel Silhouette Score	Cluster Size Distribution	Historical Accident Probability Distribution	MD
Proposed CCAE	(Loss1+Loss2)	0.2330	[11, 36, 12, 14]	[0.0909, 0.5278, 0.5000, 0.3571]	0.1265
CCAE without reference constraint	(Loss1)	0.0194	[13, 15, 33, 12]	[0.4615, 0.4000, 0.4242, 0.4167]	0.0131

Note: MD denotes the weighted mean absolute deviation of the cluster-level historical accident probabilities from the overall accident probability. Accident records were not used in model training or clustering and are reported only as an external reference.

**Table 12:** Clustering results and risk separation evaluation under different FFT truncation ratios.

Truncation Ratios	Sequence Dimension	MD	Sample Size Distribution	Historical Accident Probability Distribution	ARI vs. 10%	NMI vs. 10%
5%	90	0.107	[10, 41, 11, 11]	[0.10, 0.49, 0.55, 0.36]	0.798	0.807
6%	108	0.119	[10, 39, 12, 12]	[0.10, 0.51, 0.50, 0.33]	0.877	0.879
7%	126	0.119	[10, 39, 12, 12]	[0.10, 0.51, 0.50, 0.33]	0.877	0.879
8%	144	0.103	[10, 38, 12, 13]	[0.10, 0.50, 0.50, 0.38]	0.917	0.910
9%	162	0.115	[11, 37, 12, 13]	[0.09, 0.51, 0.50, 0.38]	0.958	0.955
10%	180	0.127	[11, 36, 12, 14]	[0.09, 0.53, 0.50, 0.36]	1.0	1.0
11%	198	0.084	[16, 10, 9, 38]	[0.50, 0.20, 0.33, 0.47]	0.525	0.386
12%	216	0.115	[11, 37, 12, 13]	[0.09, 0.51, 0.50, 0.38]	0.958	0.955
13%	234	0.142	[11, 37, 12, 13]	[0.09, 0.54, 0.50, 0.31]	0.958	0.955
14%	252	0.127	[11, 36, 12, 14]	[0.09, 0.53, 0.50, 0.36]	1.0	1.0
15%	270	0.127	[11, 36, 12, 14]	[0.09, 0.53, 0.50, 0.36]	1.0	1.0
20%	360	0.111	[11, 33, 14, 15]	[0.09, 0.52, 0.50, 0.40]	0.876	0.883
25%	450	0	[73, 0, 0, 0]	[0.43, 0, 0, 0]	-	-
30%	540	0	[73, 0, 0, 0]	[0.43, 0, 0, 0]	-	-

Note: ARI and NMI were calculated using the 10% truncation result as the baseline clustering solution. The ratios of 25% and 30% led to a single-cluster solution and were therefore treated as clustering collapse cases.

## Data Availability

The data and code are available from the corresponding author.
